# Primary care pediatricians and job satisfaction: a cross sectional study in the Lazio region

**DOI:** 10.1186/s13052-023-01511-x

**Published:** 2023-08-25

**Authors:** Martina Sapienza, Giuseppe Furia, Domenico Paolo La Regina, Valentina Grimaldi, Maria Grazia Tarsitano, Cristina Patrizi, Giovanni Capelli, Rome OMCeO Group, Antonio Magi, Stefano De Lillo, Guido Coen Tirelli, Gianfranco Damiani

**Affiliations:** 1 Department of Life Sciences and Public Health, Università Cattolica del Sacro Cuore, Largo Francesco Vito 1, 00168 Rome, Italy; 2 Hospital Management Area, Local Health Authority Roma 1, Borgo Santo Spirito 3, 00193 Rome, Italy; 3 Department of Maternal Infantile and Urological Sciences, Sapienza University of Rome, Piazzale Aldo Moro 5, 00185 Rome, Italy; 4 Directive Council of Order of Physicians and Dentists of the Province of Rome, Via Giovanni Battista de Rossi 9, 00161 Rome, Italy; 5 Primary Care Pediatrician, Local Health Authority Roma 2, Rome, Italy; 6 Department of Medical and Surgical Sciences, University “Magna Graecia”, Viale Europa, 88100 Catanzaro, Italy; 7 General Pratictioner, Legal Medicine Unit, Local Health Authority Roma 2, Rome, Italy; 8 National Center for Disease Prevention and Health Promotion, Istituto Superiore Di Sanità (ISS), Viale Regina Elena 299, 00161 Rome, Italy; 9 Department of Human Sciences, Society and Health, University of Cassino and Southern Lazio, Cassino, Italy; 10 Department of Woman and Child Health and Public Health, Fondazione Policlinico Universitario, “A. Gemelli” IRCCS, Largo Agostino Gemelli 8, 00168 Rome, Italy

**Keywords:** Primary Care Pediatricians, Job satisfaction, Pediatric Primary Care Units, Omceo Rome, Pediatricians work life balance

## Abstract

**Background:**

The Order of Physicians and Dentists of the Province of Rome aims at focusing on the satisfaction of healthcare personnel as an essential factor for the quality of medical care in the health sector. The aim of this study is to assess and prioritize the factors that can be linked to a higher or lower degree of job satisfaction in Primary Care Pediatricians (PCPs).

**Methods:**

This study is a cross sectional survey. A questionnaire was administered to all primary care pediatricians registered to the Order, exploring in particular the work activity organization, the level of satisfaction in their professional life, and the level of perceived health. A pilot activity was conducted to validate the questionnaire. Fisher exact test and ordinal logistic regression (ologit) models were used for the univariate and multivariate analysis.

**Results:**

The highest level of job dissatisfaction, in both men and women, was found to be in the practice type without any form of association; among women, it reached an even higher level for those who had their own practice at a distance of 20–40 km from their home. Women, compared to men, maintained a lower level of job satisfaction also while working in Pediatric Primary Care Units (PPCUs). In PPCUs, for the same distance, females showed a more similar pattern to males. Men working in PPCUs, regardless of distance, declared a higher degree of job satisfaction. Both men and women, working as a group pediatrician or in PPCUs, did not show a significant difference in the level of job satisfaction.

**Conclusions:**

The study contributes to a deeper understanding of the factors that may influence levels of career satisfaction in female and male PCPs. Therefore, research and interventions regarding job satisfaction should foster an organizational network connection among PCPs for their job and individual well-being, from a perspective of enhancing patient care. A major effort to improve work-life balance and career satisfaction among women is important, suggesting that interventions for improving job satisfaction could benefit from a gender-specific approach.

**Supplementary Information:**

The online version contains supplementary material available at 10.1186/s13052-023-01511-x.

## Background

Health systems must provide effective, safe and efficient health services and meet user expectations. The Italian National Health Service (INHS) is made up of three main levels of intervention: primary care, hospital care, and specialty hospital care [1]. The pediatric healthcare system is part of the INHS, and primary care physicians count among the main providers of medical assistance. (Supplementary Appendix) In recent years profound changes in primary pediatric care have taken place.

These changes are due to both the patient and the type of care required from the pediatrician. On one hand, the reduction of pediatric and neonatal mortality combined with the extension of life expectancy (attributable to improved global health conditions, availability of vaccination and antibiotic therapies), has led to an increase in the number of complex chronic patients with comorbidities. On the other hand, the pediatrician has to adapt to the new demands, both for the digitization of healthcare delivery and for the significant changes in the outpatient organization.

A combination of social, economic and technological factors (such as excessive workload or hypermedicalization, more aware and demanding parents, lack of adequate equipment, increased legal responsibility and administrative requirements) make health professionals vulnerable, influencing the services provided to their patients [2, 3]. These elements have a substantial negative impact on the professional lives of Primary Care Pediatricians (PCPs) [4]. Evidence has shown that an increased risk of medical errors is associated with burnout and significant levels of stress among physicians [5, 6].

Burnout in pediatricians was investigated in a previous study in which it was found to be associated with a more frequent engagement in disliked duties than satisfying activities. Burnout may be reduced by modifying work organization to include more involvement in professional interactions with other professionals and more varied and challenging activities. [7]

Over time there has been a growing focus on the importance of an adequate work- life balance, as well as personal and career satisfaction [8]. Satisfaction in the workplace, for physicians, is an important issue for the stability and performance of all health systems, considering that dissatisfaction and stress at work are issues shared by physicians in many countries [9–11]. Other studies have concluded that in order to increase job satisfaction and thus increase the attractiveness of pediatric primary care, new working models are required, that allow less non-medical work and intensify collaboration between pediatric physicians and professionals to dedicate more available time per patient [12].

Physicians with a high level of job satisfaction provide a higher quality service, which is more responsive to the health needs of the population [11, 13].

The Order of Physicians and Dentists of the Province of Rome (OMCeO of Rome) aims at focusing on the satisfaction of healthcare personnel as an essential factor for the quality of medical care in the health sector.

To our knowledge, there has been little research on job satisfaction of PCPs; the present study, which could be easily replicated by other researchers, provides an initial overview of the possible reasons for professional dissatisfaction, that might enable stakeholders to identify targeted interventions.

The aim of this work is to assess and prioritize the factors that could improve and optimize the degree of job satisfaction in PCPs.

## Methods

### Study design

This study was based on a cross-sectional survey performed through the administration of an anonymous questionnaire, developed by a focus group promoted by the OMCeO of Rome and intended for all PCPs registered to said Order of Rome. For bibliographic research, relevant electronic databases for scientific literature were queried. The questionnaire was designed and written in Italian. The institutional website of OMCeO was used to provide a rapid and easy access to all users interested and invited to fill out the questionnaire.

The questionnaire consisted of 28 questions (Supplementary Questionnaire).

At the end of the questionnaire, socio-demographic variables of participants (gender, age, citizenship, marital status, fragile individuals taken in charge) were investigated. Variables were selected considering the domains investigated by I Perez-Ciordia [14] et al and AJ Starmer et al [15], although including some specific variables related to the peculiarities of the INHS. Questions included from a minimum of two to a maximum of five mutually exclusive responses. A pilot activity was initially conducted to test the questionnaire and finalize the questions. The questionnaire was administered electronically to PCPs who are members of the OMCeO of Rome through the Italian Federation of Pediatricians (FIMP) Rome and Lazio. The final survey began on July 31st, 2020, and ended on February 10th, 2021.

### Study variables

The main dependent variable evaluated was "job satisfaction," expressed as: 1-Definitely yes, 2-More yes than no, 3-More no than yes, 4-Definitely no

The following exposure variables were assessed (independently and together): Home to workplace distance, Gender, Practice type, Age class, Caregiving.

### Statistical analysis

A descriptive statistical analysis was performed to map the population profiles studied and the level of satisfaction of professionals. All statistical analyses were performed using Stata version 16. Fisher exact test was used for the univariate analysis and proportional odds/ordinal logistic regression (ologit) models were performed to evaluate the univariate and multivariate association or confounding effect of distance from home to work, practice type, gender, age and being caregiver of children or elderly people on the 4-level ordinal scale of job satisfaction.

## Results

In the pilot phase, the draft questionnaire was submitted to 15 PCPs of different ages and experience. Suggestions and responses from questionnaire responders resulted in the rewording of some questions. In the study phase, the questionnaire was sent to 772 pediatricians, filled out by 243 pediatricians, of which 158 compiled all answers (20.5% of respondents) whereas 85 pediatricians did not complete the whole questionnaire. Table 1 shows the characteristics of the responders. The most represented gender was the female one (74.1%), and more than half (50.6%) were over 60 years old, with more than 25 years of career (55.7%). 29.8% lived outside the Province of Rome, but the home-work distance did not exceed 20 km in 75.9% of respondents, with 39.2% of respondents working within 5 km of their home. In addition, 77.2% of responding pediatricians reported having children or other people to care for (elderly, disabled, etc.). Concerning the type of work they conducted, 73.4% worked in associations of PCPs—called Pediatric Primary Care Units (PPCUs) -, organized either in a single workplace or connected to a network, so as to provide continuous pediatric primary care to all the patients of the associated pediatricians. In order to ensure the best service, the patient records are shared with each component of the PPCUs. 15.8% worked alone, without any form of association, and the remaining 10.8% worked as group pediatricians. The overall prevalence of job satisfaction in our sample was 84.51%.

**Table 1:** Characteristics of respondents

**Gender**	**F**	**M**		
	**117**	41		
	***74.1%***	*25.9%*		
**Age**	** < 50**	**50–60**	**> 60**	
	18	60	**80**	
	*11.4%*	*38.0%*	***50.6%***	
**Years of pediatric career**	** < 5**	**5–10**	**11–25**	**> 25**
	16	15	39	**88**
	*10.1%*	*9.5%*	*24.7%*	***55.7%***
**Practice placement in the city**	**Historical center**	**Within Great Ring Junction**	**Outside Great Ring Junction**	**Province**
	7	**70**	34	47
	*4.4%*	***44.3%***	*21.5%*	*29.8%*
**Home to workplace distance**	** < 5 km**	**5–20 km**	**20–40 km**	** > 40 km**
	**62**	58	22	16
	***39.2%***	*36.7%*	*13.9%*	*10.1%*
**Caregiving—Children or other people to take care of (Elderly, Disabled)**	**Yes**	**No**		
	**122**	36		
	***77.22%***	*22.78%*		
**Work activity organization (Practice type)**	**PPCU**	**Solo professional practitioner**	**Associated medicine**	
	**116**	25	17	
	***73.42%***	*15.82%*	*10.76%*	

### Univariate analysis – Fisher’ exact test

The factors "distance between home and workplace", "gender: male" and "practice type" appeared to be associated with job satisfaction (*p* = 0.026, p = 0.031 and *p* = 0.001, respectively, at Fisher exact test) in the univariate analysis. Instead, age and having children or other persons to care for, did not appear to be related to job satisfaction level (*p* = 0.195 and 0.792, respectively) Table 2.

**Table 2:** Univariate analysis

**Variable**	Job satisfaction level*					**Fisher's exact p**
***Distance***	*Definitely not*	*More no than yes*	*More yes than no*	*Definitely yes*		
< 5 km	0	3	27	30	60	0.026
5–20 km	4	7	28	19	58	
20–40 km	1	6	10	5	22	
> 40 km	1	2	9	3	15	
***Gender***						
M	5	16	60	35	116	0.031
F	1	2	14	22	39	
***Practice type***						
Solo professional practitioner	1	8	11	3	23	0.001
Associated Medicine	0	4	6	7	17	
PPCUs	5	6	57	47	115	
***Age***						
< 50 yrs	1	3	5	9	18	0.097
50–60 yrs	4	8	31	15	58	
> 60 yrs	1	7	38	33	79	
***Caregiver***						
No	1	5	14	14	34	0.792
Yes	5	13	60	43	121	
***TOTALS***	6	18	74	57	155	

^*^3 respondents answered “don’t know” to the question about job satisfaction level

### Univariate and Multivariable modeling

The three variables that resulted to be significant in the univariate analyses (gender, practice type, and distance in km between the workplace and home), were found to be statistically significant both in univariate and multivariable ordered logit models and appeared to have an independent effect on the primary outcome variable (job satisfaction). The results of the univariate models, a model with all 5 variables and a more parsimonious but equally predictive model with only 3 variables (Gender, Practice Type and Home to workplace distance) are shown in detail in Table 3. The ordinal logistic regression (ologit) models confirmed the non-significance of the covariates 'age and caregiving' (having children or other people to take care of), which appear to have nor an effect on job satisfaction (since the C.I. of the coefficents always includes the value of 0) neither a confounding effect on the other variables (since the coefficents on all the other 3 variables do not change or change in a negligible way in the 5-variables model).

**Table 3:** Results of proportional odd models, Univariate and Multivariable (3 variables and 5 variables models)

**Model**	**Univariate**			**Multivariable—3 variables**			**Multivariable—5 variables**		
***Home-workplace distance***	*Beta*	*CI 95%*	*p*	*Beta*	*CI 95%*	*p*	*Beta*	*CI 95%*	*p*
< 5 km	baseline			baseline			baseline		
5–20 km	-0.877	(-1.580; -0.175)	0.014	-0.920	(-1.645; -0.194)	0.013	-0.895	(-1.630; -0.159)	0.017
20–40 km	-1.486	(-2.449; -0.524)	0.002	-1.597	(-2.576; -0.617)	0.001	-1.429	(-2.457; -0.401)	0.006
> 40 km	-1.273	(-2.352; -0.193)	0.021	-0.662	(-1.822; 0.497)	0.263	-0.732	(-1.963; 0.498)	0.243
/cut1	-4.015	(-4.971; -3.060)		-4.338	(-5.401; -3.275)		-4.200	(-5.424; -2.977)	
/cut2	-2.456	(-3.097; -1.814)		-2.655	(-3.405; -1.904)		-2.536	(-3.510; -1.557)	
/cut3	-0.065	(-0.552; 0.423)		0.061	(-0.507; 0.631)		0.235	(-0.617; 1.088)	
***Gender***									
F	baseline			baseline			baseline		
M	1.074	( 0.353; 1.795)	0.004	1.308	(0.537; 2.080)	0.001	1.400	(0.616; 2.184)	< 0.001
/cut1	-3.028	(-3.852; -2.204)		*-4.338*	*(-5.401; -3.275)*		*-4.200*	*(-5.424; -2.977)*	
/cut2	-1.499	(-1.952; -1.047)		*-2.655*	*(-3.405; -1.904)*		*-2.536*	*(-3.510; -1.557)*	
/cut3	0.830	( 0.442; 1.218)		*0.061*	*(-0.507; 0.631)*		*0.235*	*(-0.617; 1.088)*	
***Practice type***									
PPCUs	baseline			baseline			baseline		
Solo professional practitioner	-1.523	(-2.396; -0.650)	0.001	-1.780	(-2.693; -0.868)	< 0.001	-1.877	(-2.816; -0.937)	< 0.001
Associated Medicine	-0.280	(-1.288; 0.728)	0.587	-0.357	(-1.387; 0.673)	0.497	-0.263	(-1.318; 0.791)	0.625
/cut1	-3.661	(-4.552; -2.770)		*-4.338*	*(-5.401; -3.275)*		*-4.200*	*(-5.424; -2.977)*	
/cut2	-2.076	(-2.606; -1.547)		*-2.655*	*(-3.405; -1.904)*		*-2.536*	*(-3.510; -1.557)*	
/cut3	0.338	(-0.024; 0.700)		*0.061*	*(-0.507; 0.631)*		*0.235*	*(-0.617; 1.088)*	
***Age***									
> 60	baseline			-			baseline		
50–60	-0.738	(-1.386; -0.090)	0.026	-			-0.398	(-1.137; 0.341)	0.291
< 50	0.008	(-1.017; 1.034)	0.988	-			0.615	(-0.482; 1.713)	0.272
/cut1	-3.545	(-4.436; -2.654)					*-4.200*	*(-5.424; -2.977)*	
/cut2	-2.016	(-2.568; -1.464)					*-2.536*	*(-3.510; -1.557)*	
/cut3	0.283	(-0.145; 0.710)					*0.235*	*(-0.617; 1.088)*	
***Caregiving***									
Yes	baseline			-			baseline		
No	0.121	(-0.607; 0.849)	0.745	-			0.242	(-0.573; 1.057)	0.561
/cut1	-3.187	(-4.017; -2.357)					*-4.200*	*(-5.424; -2.977)*	
/cut2	-1.672	(-2.132; -1.212)					*-2.536*	*(-3.510; -1.557)*	
/cut3	0.568	(0.206; 0.930)					*0.235*	*(-0.617; 1.088)*	

**Fig. 1:**
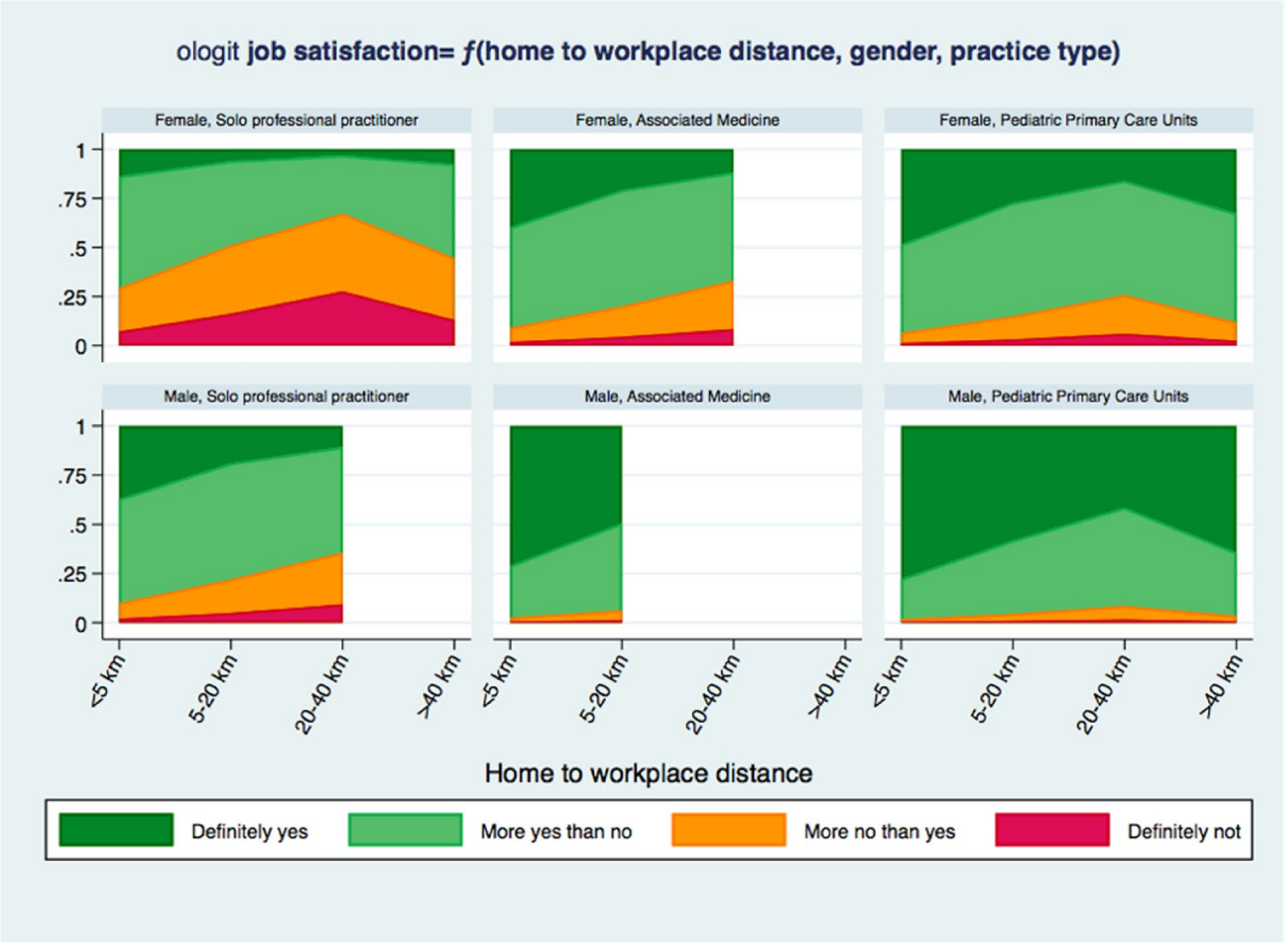
Probability of the level of job satisfaction predicted by the ordinal logistic regression model

Therefore the more parsimonious 3-variables model was chosen as the one which better summarizes the results of the study: the probability of a different level of job satisfaction predicted by the 3-variable model for different values of the variable included in it is shown in Fig. 1, to get a better understanding of the results of the ologit model Fig. 1.

In particular, in this model male gender is positively correlated with a higher degree of job satisfaction than female gender showing an expected value for the coefficient of 1.31 (0.54 ÷ 2.08, 95% CI), significantly higher that 0 at a *p* value=0.001. On the contrary, working alone, with no form of association, worsens the perception of being satisfied of the own’s job compared to working in PPCUs, showing a very significant (*p*<0.001) negative coefficient of -1.78 (-2.69 ÷ -0.87, 95% CI). Moreover, living at a higher distance from the workplace reduced satisfaction levels compared to a distance of less than 5 km: in particular, for a distance between 5-20 km the model showed a negative coefficient of -0.92 (-1.65 ÷ -0.19, 95% CI), with a p value=0.013, and for a distance of 20-40 km the model showed a negative coefficient of -1.60 (-2.58 ÷ -0.62, 95% CI) with a *p* value= 0.001.

## Discussion

This study highlighted that the highest level of job dissatisfaction, in both men and women, has a higher probability of being found in professionals who operate in the type of medical practice without any form of association; in women, it even reaches a higher level (“more no than yes”) in those who have a workplace at a distance of 20-40 km from their home. Women, compared to men, maintain a lower probabilty of high job satisfaction (“definitely yes”) even while working in PPCUs. In PPCUs, for the same distance, females show a pattern more similar to males. Men working in PPCUs, instead, declare a higher degree of job satisfaction regardless of distance. In both men and women, whether they work as group pediatricians or in PPCUs, no significant difference in the level of job satisfaction is detectable.

Based on Porter’s research [16], Value-Based Healthcare is a framework for restructuring healthcare systems around the globe: the overall goal is to establish a continuous improvement process that places the patient figure at the center of the care pathway, in order to deliver high quality health care and improve the patients’ experience. According to Berwick et al. 2008 [17], improving the health care system requires the successful concurrent achievement of three purposes: improving the experience of care, bettering population health, and reducing per capita costs of healthcare. In 2014, Bodenheimer et al. [18], expanded to a Quadruple Aim, adding the goal of improving the work life of healthcare providers, including clinicians and staff. Dissatisfied physicians are two to three times more likely to quit practice, worsening the increasing shortage of primary care physicians and hindering the possibility of obtaining and maintaining an healthy population [18]; therefore, the quality of services in a healthcare system is related to the level of satisfaction of its professionals [14]. In this perspective, this study showed that the PCPs’ profile with the lowest level of career satisfaction are women who work alone and away from home, while men would rather work in PPCUs than practice alone as well; therefore it could be useful to promote an organizational connection between the PCPs to improve their working and individual well-being, in a perspective of enhancing a value-based patient care.

The study by Starmer et al [15] corroborates our findings, showing that female pediatricians are more likely to report struggling with work-life balance and less likely to report job satisfaction than men. Given the higher proportion of women in pediatrics in Italy, a major effort to improve work-life balance and career satisfaction among them is of vital importance, suggesting that planning interventions for improving job satisfaction could benefit from a gender-specific approach. The study presents several strengths and limitations, including that all data described in this article are self-reported and cross-sectional, and therefore do not allow an understanding of the temporal nature of how factors associated with PCPs’ job satisfaction might have an impact over time. In addition, more than half (50.6%) of the pediatricians who completed the questionnaire were over 60 years old, meaning that our sample of respondents may be biased, as a higher number of younger respondents could influence the results differently. . The fact that there are very few pediatricians under the age of 50 and the aging of this professional category is a critical issue to be investigated and as such it raises reflections, especially it poses a challenge in pediatric care programming also with a perspective of meeting the new organizational model of the network of territorial health care as disciplined by Ministerial Decree n. 77 of June 22, 2022. Not all the dimensions that can affect job satisfaction were investigated, e.g. complexity of patients, mental health status of pediatricians. Nonetheless, the accessibility of the survey, which can be filled in electronically from any device, has made the sample size representative and the relationship is very close and consistent, since the p values are very low. Seeing that a survey on job satisfaction among primary care pediatricians that are registered with the the Omceo of Rome has never been conducted, starting from these results and specifically analyzing the critical issues that emerged from the survey could allow to conduct further research by extending the issues to other areas (chronic disease management, low professionalism, professional development, resiliency training, healthcare organization, amount of working hours and workload related to the number of patients, carrying out administrative tasks, provider-perceived safety culture, continuing medical education, collaboration with other healthcare providers, access to specialized services for patients, lack of time for patient consultation). Particular attention should be paid to mental health, as it is an important indicator of the psychological state of employees, which directly affects their perception of job satisfaction [19]. Based on these results, the work could also be extended to other medical specialties to highlight new critical issues for other categories of physicians and carry out improvement actions on what emerged. In addition, the Omceo of Rome could propose solutions at an institutional level to optimize work reorganization also from a gender perspective, and to consider combining traditional medical practice with telemedicine, which is an excellent solution to ensure care and assistance for children even at a distance, facilitating pediatrician-patient communication, the exchange of clinical information, and better management of the pediatrician's work even from home.

## Conclusion

The presented study contributes to a deeper understanding of the factors that influence levels of career satisfaction of female and male PCPs. Therefore, research and interventions regarding job satisfaction should foster an organizational work connection among PCPs for their work and individual well-being from a perspective of enhancing patient care. PPCUs ensure continuous pediatric primary care for all patients of the associated pediatricians, through sharing of patient records in the network by all PPCUs pediatricians, allow uniformity and continuity of pediatric primary care health services, and capillarity of health information with improved professional satisfaction for the pediatricians. Further studies could be conducted comparing our results with those of PCPs living in a nonmetropolitan city with shorter distances. In addition, the study could be expanded by also focusing on the mental health of PCPs, which could significantly affect job satisfaction, and export this study model to other categories of general and specialist physicians

### Supplementary Information

**Additional file 1** Supplementary Appendix.

**Additional file 2** Supplementary Questionnaire.

## Data Availability

The datasets used and/or analysed during the current study are available from the corresponding author on reasonable request.

## Abbreviations

PCPs: Primary Care Pediatricians
OMCeO of Rome: Order of Physicians and Dentists of the Province of Rome
FIMP: Italian Federation of Pediatricians
PPCUs: Pediatric Primary Care Units
