# Supplementary material for: Primary care pediatricians and job satisfaction: a cross sectional study in the Lazio region
Source: Ital J Pediatr. 2023 Aug 25;49:104. doi: 10.1186/s13052-023-01511-x (PMC10463623; doi:10.1186/s13052-023-01511-x)
Supplement: Supplementary file 1 — Additional file 1 Supplementary Appendix. [file 13052_2023_1511_MOESM1_ESM.docx]

Studies of European countries reveal profound differences in the organization of children’s (nonhospital) first-contact services. Three main models exist, which are based on whether primary care general physicians, primary care pediatricians (PCPs), or combinations of both are primarily responsible for care. In Italy, pediatric primary care up to 6 years of age is provided exclusively by PCPs.

**BOX**

**Primary Care**

The pediatric health care system in Italy is part of the national health system. It is made up of 3 main levels of intervention: first access/primary care, secondary care/hospital care, and tertiary care based on specialty hospital care.

Primary care includes general first-access care for children and adolescents (0-16 years), which is provided by PCPs paid through a state collective agreement. It is organized in a national “family pediatrics” network. Such a system was established in 1981. Since then, the Italian National Health Service has provided pediatric primary care to children through the PCPs, who are commonly called family pediatricians. The Italian Public Health Care System requires that all children have an identified primary care provider, depending on the patient’s age. Italian pediatricians related to the Public Health Care System work in their own private offices, providing primary care of patients from birth to 16 years of age and are compensated under a capitation system, based on the number of children registered with each PCP. Pediatricians working for the Public Health Care system are usually the sole patient entrance to public secondary and tertiary care in range of 0-6 years of age, and parents can choose between a pediatrician and a GP for their children who are between 6 and 16 years of age.

Pediatricians who wish to work with other colleagues, can do so in two ways*: Pediatric Primary Care Units (PPCUs) and Associated Medicine.*

UCPPs are forms of association where patient management is shared, guaranteeing peer comparison and a medical presence throughout the day in a single medical office.

On the contrary, Associated Medicine consists of a care model based on the functional interaction between several family pediatricians operating in their respective offices through telematic tools.
