# Supplementary material for: Primary care pediatricians and job satisfaction: a cross sectional study in the Lazio region
Source: Ital J Pediatr. 2023 Aug 25;49:104. doi: 10.1186/s13052-023-01511-x (PMC10463623; doi:10.1186/s13052-023-01511-x)
Supplement: Supplementary file 2 — Additional file 2 Supplementary Questionnaire. [file 13052_2023_1511_MOESM2_ESM.docx]

PEDIATRI DI LIBERA SCELTA - QUESTIONARIO DI SODDISFAZIONE

Gentili Colleghi,

data l'importanza delle cure primarie per i sistemi sanitari e la salute della popolazione, sembra cruciale identificare i fattori che contribuiscono alla qualità delle stesse.

Secondo alcune ricerche la riduzione del benessere professionale e della soddisfazione lavorativa dei medici di assistenza primaria potrebbero compromettere le competenze

professionali, gli aspetti relazionali con i pazienti e quindi anche la soddisfazione di questi ultimi relativamente alle cure ricevute.

Si prevede che i continui cambiamenti nel sistema sanitario avranno un impatto sostanziale sulla vita personale e professionale dei pediatri. I tassi elevati di stress e burnout tra i medici sono ben documentati, e hanno dimostrato di essere associati ad un aumentato rischio di errori medici.

Con un sistema sanitario in continuo mutamento, bisogni assistenziali dei pazienti sempre più complessi e l’avanzare della tecnologia in ambito medico, si rende necessario porre maggiore attenzione all'importanza di un adeguato equilibrio tra lavoro e vita privata e alla soddisfazione personale e di carriera.

I fattori di stress emotivo sul posto di lavoro a lungo termine possono generare un'assistenza non ottimale ai pazienti e sono stati considerati potenziali fattori di rischio di burnout e abbandono della professione medica.

OMCeO Roma ha avviato uno studio sulla soddisfazione lavorativa e sulla qualità della vita dei Pediatri di Libera Scelta di Roma. Il questionario è anonimo e l’adesione allo studio è volontaria.

Il Presidente

(Dott. Antonio Magi)

Ci sono 28 domande in questa indagine.

# Pediatria di libera scelta


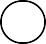


Sei un pediatra di libera scelta? *

 Scegliere solo una delle seguenti voci Scegli **solo una** delle seguenti:

Si
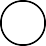
 No

Attività Lavorativa


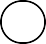


Posizione del tuo Studio? *

 Scegliere solo una delle seguenti voci Scegli **solo una** delle seguenti:

Centro storico


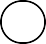
 Comune di Roma, dentro il raccordo anulare
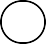
 Comune di Roma, fuori dal raccordo anulare
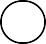
 Nella Provincia di Roma


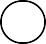


Quanto dista casa dal luogo di lavoro? *

 Scegliere solo una delle seguenti voci Scegli **solo una** delle seguenti:


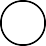
 < 5 Km

5 - 20 km


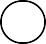
 20 - 40 km


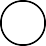
 > 40 km


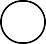


Da quanto tempo lavori come PLS? *

 Scegliere solo una delle seguenti voci Scegli **solo una** delle seguenti:

< 5 anni


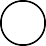
 5 - 10 anni


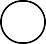
 11 - 25 anni


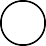
 > 25 anni


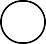


Numero Assistiti? *

 Scegliere solo una delle seguenti voci Scegli **solo una** delle seguenti:

Massimale
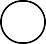
 > 600


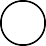
 600 - 300


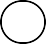
 < 300


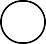


Numero Assistiti stranieri? *

 Scegliere solo una delle seguenti voci Scegli **solo una** delle seguenti:

Nessuno


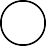
 1/3 degli assistiti


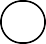
 1/3 - 2/3 degli assistiti
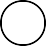
 > 2/3 degli assistiti


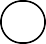


Ti ritieni soddisfatta/o della gestione del tuo studio? *

 Scegliere solo una delle seguenti voci Scegli **solo una** delle seguenti:

Si
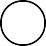
 No


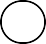


Tipologia di lavoro? *

 Scegliere solo una delle seguenti voci Scegli **solo una** delle seguenti:

Da solo, senza nessuna forma associativa
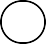
 UCPP


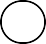
 Pediatria di gruppo


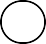


Ti ritieni soddisfatta/o relativamente a tempi, orari vistia, accesso dei pazienti? *

 Scegliere solo una delle seguenti voci Scegli **solo una** delle seguenti:

Si
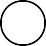
 No


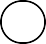


Come accedono gli assistiti? *

 Scegliere solo una delle seguenti voci Scegli **solo una** delle seguenti:


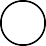
 Con appuntamento Accesso libero


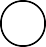
 Misto


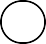


Somministri i vaccini presso il tuo studio? *

 Scegliere solo una delle seguenti voci Scegli **solo una** delle seguenti:

Si
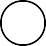
 No


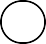


Quanti vaccini somministri presso il tuo studio? *

Rispondere solo se le seguenti condizioni sono rispettate:

La risposta era 'Si' Alla domanda '11 [L10]' (Somministri i vaccini presso il tuo studio?)

 Scegliere solo una delle seguenti voci Scegli **solo una** delle seguenti:

1 - 10 dosi di vaccino antinfluenzale /anno
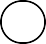
 11 - 20 dosi all'anno


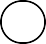
 21 - 50 dosi al' anno


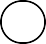
 51 - 100 dosi all'anno


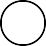
 > 100


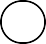


Possiedi un numero dedicato ai pazienti e uno privato? *

 Scegliere solo una delle seguenti voci Scegli **solo una** delle seguenti:

Si
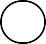
 No


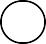


Tieni il telefono acceso anche sabato e festivi? *

 Scegliere solo una delle seguenti voci Scegli **solo una** delle seguenti:

Si
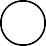
 No


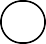
 a volte


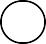


Come giudicheresti la tua attività lavorativa? *

 Scegliere solo una delle seguenti voci Scegli **solo una** delle seguenti:

Eccessivamente frenetica
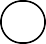
 Saltuariamente frenetica


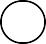
 Piena ma nei limiti del ragionevole


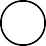
 Tranquilla

Grado di soddisfazione


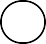


L'equilibrio vita privata-lavoro è buono *

 Scegliere solo una delle seguenti voci Scegli **solo una** delle seguenti:

Sono pienamente d'accordo
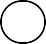
 Più si che no


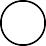
 Non sono per niente d’accordo


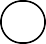
 Più no che si
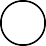
 Non saprei


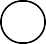


Vivo una condizione di fort stress a lavoro *

 Scegliere solo una delle seguenti voci Scegli **solo una** delle seguenti:

Sono pienamente d’accordo
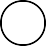
 Più si che no


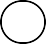
 Non sono per niente d’accordo


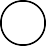
 Più no che si
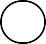
 Non saprei


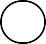


Tutto sommato, sono soddisfatto della mia carriera di Pediatra

*

 Scegliere solo una delle seguenti voci Scegli **solo una** delle seguenti:

Sono pienamente d’accordo
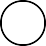
 Più si che no


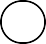
 Non sono per niente d’accordo


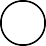
 Più no che si
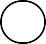
 Non saprei


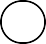


Complessivamente, quanto ritieni soddisfacente la tua vita privata?

*

 Scegliere solo una delle seguenti voci Scegli **solo una** delle seguenti:

Totalmente
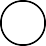
 Molto


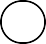
 Abbastanza


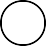
 Poco


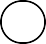
 Per niente

Salute


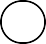


Come reputi il tuo stato di salute generale?

*

 Scegliere solo una delle seguenti voci Scegli **solo una** delle seguenti:

Eccellente
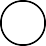
 Buono


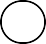
 Mediocre


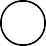
 Pessimo


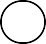


Hai vissuto eventi negativi negli ultimi 12 mesi (ad es. lutti o malattie gravi in famiglia,divorzi, difficoltà finanziarie)?

*

 Scegliere solo una delle seguenti voci Scegli **solo una** delle seguenti:

Nessuno
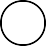
 ≥ 1


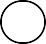


Ore di sonno in una giornata tipo

*

 Scegliere solo una delle seguenti voci Scegli **solo una** delle seguenti:

≥ 7


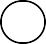
 < 7


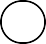


Ti sei sentita/o depresso nell'ultimo anno?

*

 Scegliere solo una delle seguenti voci Scegli **solo una** delle seguenti:


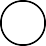
 Sempre Spesso


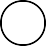
 Qualche volta
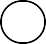
 Mai

Generalità


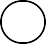


Genere *

 Scegliere solo una delle seguenti voci Scegli **solo una** delle seguenti:

M
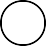
 F


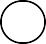


Cittadinanza *

 Scegliere solo una delle seguenti voci Scegli **solo una** delle seguenti:

Italiana
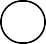
 Straniera


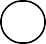


Stato civile *

 Scegliere solo una delle seguenti voci Scegli **solo una** delle seguenti:


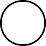
 Nubile/Celibe Sposato/Convivente


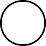
 Divorziato/Separato
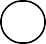
 Vedovo/a


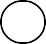


Quanti hanni hai?

*

 Scegliere solo una delle seguenti voci Scegli **solo una** delle seguenti:

< 30


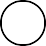
 30 - 40


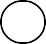
 40 - 50


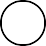
 50 - 60


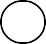
 > 60


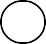


HAI FIGLI O ALTRE PERSONE DI CUI DEVI PRENDERTI CURA (ANZIANI, DISABILI)?

Hai figli o altre persone di cui devi prenderti cura (anziani, disabili)?

*

 Scegliere solo una delle seguenti voci Scegli **solo una** delle seguenti:

Si
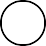
 No

Grazie per aver dedicato il tuo tempo al nostro questionario

Questa indagine è anonima.

La registrazione delle risposte fornite all'indagine non è relativa a informazioni che consentano l'identificazione del rispondente, a meno che qualche domanda del questionario non la chieda esplicitamente.

Se è stato usato un codice identificativo per accedere a quest'indagine, questo codice non sarà registrato assieme alle risposte fornite. Il codice identificativo è gestito in un database diverso e viene aggiornato soltanto per indicare se è stata completata (o no) l'indagine.

Non c'è nessun modo per abbinare i codici identificativi alle risposte dell'indagine.

09.03.2021 – 09:08

Inviare il questionario.

Grazie per aver completato il questionario.
